# Stochasticity and Randomness in Community Assembly: Real or As-If?

**DOI:** 10.1128/mSystems.00938-21

**Published:** 2021-09-07

**Authors:** Joseph D. Madison

**Affiliations:** a Department of Biology, University of Massachusetts Boston, Boston, Massachusetts, USA; Southern Medical University

## LETTER

In a recent paper by Ma (1), results of a modified multisite neutral model analysis of community assembly inform a provocative claim that stochasticity correlates with a phylogenetic timeline. Two related results are that humans exhibit complete stochastic neutrality in microbial community assembly and that these results are of significance in understanding assembly processes. Consequently, Ma says: “[I]f the stochastic (random) forces are indeed significant in maintaining microbiome structures, the design of human interventions to maintain or restore microbiome structures … cannot ignore this important, innate aspect of the animal/human microbiomes.”

While I concur that the understanding of stochastic processes is of great significance, I would urge that Ma and others use caution in neutral model interpretations of stochastic processes. This especially applies in considering model-stochastic import to real-stochastic phenomena of related randomness or causality-impoverished indeterminacy. To briefly clarify the semantic distinctions as I use them here: stochasticity deals with process, whereas randomness may deal with the product of process. This product-process distinction has support à la Tukey (2). A more recent paper outlines these taxonomies with a similar distinction and also asserts randomness as unpredictability (3).

To the main point: work such as that by Vellend et al. (4) acknowledge the philosophical issues present in neutral models of stochasticity in community ecology. However, I believe this and similar accounts understate the problem in claiming a “with-respect-to sense” of stochasticity that “may have great utility in ecology,” implying it is “usefully considered stochastic”. This telling confers an as-if conception of processual stochasticity. Alternatively, one could hold an epistemic claim of stochastic processes generating real randomness. This is an important distinction: as-if randomness implies some causal mechanism that might possibly be understood (new technologies, theories, etc.). Real randomness is aptly described by Hellman (5)—a physical process with an outcome that is “not causally determined”. Importantly, Hellman questions the mapping of set-theoretic randomness to physical or real randomness. He also expresses the sentiment that if real randomness exists, there is nothing more for us to directly explicate. A need for delineation is therefore acute where modeling or other mathematical stochasticity suggests randomness that is not necessarily in the real world. For example, take the three essential properties of randomness from Kolmogorov and Upsenkii (6) for a set (Ω) of infinite sequences (σ) on a Bernoulli measure, of which the von Mises conception of stochasticity is only one part (the others being chaos and typicality). While σ may be defined random as such, this tells us nothing of the real-world mapping of σ to a physical process or product that has randomness in the sense of real-world indeterminate causality.

I do not know which of these conceptions is better, and I will not attempt to tackle their ontological or metaphysical status. Nevertheless, it seems to me that the epistemic and explanatory consequences of using real or as-if conceptions—in the milieu of stochasticity, randomness, and indeterminacy—are dramatically different. Therefore, this distinction must be addressed when speaking to neutral theory models of microbial community assembly.
